# Draft Genome Sequence of *Staphylococcus epidermidis* Clinical Strain GOI1153754-03-14 Isolated from an Infected Knee Prosthesis

**DOI:** 10.1128/genomeA.00378-17

**Published:** 2017-05-18

**Authors:** Marta Bottagisio, Alessio Soggiu, Arianna B. Lovati, Marco Toscano, Cristian Piras, Carlo L. Romanò, Luigi Bonizzi, Paola Roncada, Lorenzo Drago

**Affiliations:** a Cell and Tissue Engineering Laboratory, IRCCS Galeazzi Orthopedic Institute, Milan, Italy; b Department of Veterinary Medicine (DiMeVet), University of Milan, Milan, Italy; c Department of Biomedical Science for Health, University of Milan, Milan, Italy; d Center for Reconstructive Surgery of Osteoarticular Infections, CRIO, IRCCS Galeazzi Orthopedic Institute, Milan, Italy; e Istituto Sperimentale Italiano Lazzaro Spallanzani, Cremona, Italy; f Laboratory of Clinical Chemistry and Microbiology, IRCCS Galeazzi Orthopedic Institute, Milan, Italy

## Abstract

We announce the draft genome sequence of *Staphylococcus epidermidis* clinical strain GOI1153754-03-14, isolated from an infected orthopedic prosthesis. The reported genomic sequence will provide valuable information concerning the mechanisms of the biofilm formation on metallic implants.

## GENOME ANNOUNCEMENT

Implant-related infections are the most severe complications following joint arthroplasty and represent a socioeconomic burden. Consequently, it is important to pore over the interaction between pathogens and the host immune response along with the mechanisms leading to prosthetic infections (1). This complex process starts with bacterial contamination, adhesion, and biofilm formation on the implant surface, thus conferring to bacteria a protection from both the host immune system and antibiotics (2).

Among several pathogens involved in implant-related infections, staphylococci account for 82.3% of clinically isolated bacteria. In the presence of medical devices, *S. aureus* infection accounts for 31.7% of all isolates, while *S. epidermidis* accounts for 39% (3).

*Staphylococcus epidermidis* is a commensal Gram-positive, coagulase negative pathogen responsible for delayed, low-grade nosocomial infections characterized by the absence of specific clinical signs and hardly distinguishable from aseptic prosthetic loosening (4, 5).

In this work, we announce the draft genome sequence of *S. epidermidis* clinical strain GOI1153754-03-14 derived from an infected knee prosthesis of a patient that underwent implant revision at the Center for Reconstructive Surgery of Osteoarticular Infections (CRIO, IRCCS Galeazzi Orthopedic Institute, Milan, Italy), and isolated at the Laboratory of Clinical Chemistry and Microbiology (IRCCS Galeazzi Orthopedic Institute, Milan, Italy).

The antimicrobial susceptibility and MIC of this strain were carried out on a Vitek2 system (Biomérieux, Craponne, France), displaying resistance to benzylpenicillin (MIC ≥ 0.5 μg/ml), oxacillin, cefazolin, rifampin, and levofloxacin (MIC ≥ 4 μg/ml) (6).

Genomic DNA from bacterial culture was extracted using a bacterial genomic DNA isolation kit (Norgen Biotek Corp., Thorold, ON, Canada) according to the manufacturer’s guidelines, and quantified through the NanoDrop 2000 UV-Vis Spectrophotometer (Thermo Fisher Scientific, Inc., Waltham, MA, USA).

Libraries were prepared by means of the ThruPLEX DNA-seq (Rubicon Genomics, Ann Arbor, MI, USA). The isolate was sequenced on the Illumina MiSeq platform through the MiSeq reagent kit v3 (600-cycles) to produce 300 bp paired-end reads (Illumina, Inc., San Diego, CA, USA).

The outputs were quality-trimmed using ERNE-Filter (7) into 51 contigs (average = 50,720.6 Mb; max = 280,473 Mb; min = 633 Mb) with 396× fold average coverage. The combined length of the contigs was 2,586,753 bp with a G+C content of 31.84% and an *N*_50_ value of 7 bp. Gene annotations were performed thorough the RAST software (8), resulting in a total of 2,467 protein-encoding genes and 64 RNAs (55 tRNAs and 9 rRNAs).

Since the ability of *S. epidermidis* GOI1153754-03-14 to colonize implants and to cause septic nonunions was already validated in a recent *in vivo* study (6), the deposition of the draft genome sequence will enable deeper insight into the mechanisms of prosthetic joint infections.

### Accession number(s).

This whole-genome shotgun project has been deposited in DDBJ/ENA/GenBank under the accession no. FWCG01000000. The version described in this paper is the first version, FWCG01000000.
